# Novel (p)ppGpp Binding and Metabolizing Proteins of *Escherichia coli*

**DOI:** 10.1128/mBio.02188-17

**Published:** 2018-03-06

**Authors:** Yong Zhang, Eva Zborníková, Dominik Rejman, Kenn Gerdes

**Affiliations:** a Department of Biology, University of Copenhagen, Copenhagen, Denmark; b Institute of Organic Chemistry and Biochemistry, Czech Academy of Sciences v.v.i., Prague, Czech Republic; University of Michigan-Ann Arbor

**Keywords:** (p)ppGpp, DRaCALA, GTPase, Nudix, multidrug tolerance, persistence

## Abstract

The alarmone (p)ppGpp plays pivotal roles in basic bacterial stress responses by increasing tolerance of various nutritional limitations and chemical insults, including antibiotics. Despite intensive studies since (p)ppGpp was discovered over 4 decades ago, (p)ppGpp binding proteins have not been systematically identified in *Escherichia coli*. We applied DRaCALA (differential radial capillary action of ligand assay) to identify (p)ppGpp-protein interactions. We discovered 12 new (p)ppGpp targets in *E. coli* that, based on their physiological functions, could be classified into four major groups, involved in (i) purine nucleotide homeostasis (YgdH), (ii) ribosome biogenesis and translation (RsgA, Era, HflX, and LepA), (iii) maturation of dehydrogenases (HypB), and (iv) metabolism of (p)ppGpp (MutT, NudG, TrmE, NadR, PhoA, and UshA). We present a comprehensive and comparative biochemical and physiological characterization of these novel (p)ppGpp targets together with a comparative analysis of relevant, known (p)ppGpp binding proteins. Via this, primary targets of (p)ppGpp in *E. coli* are identified. The GTP salvage biosynthesis pathway and ribosome biogenesis and translation are confirmed as targets of (p)ppGpp that are highly conserved between *E. coli* and *Firmicutes*. In addition, an alternative (p)ppGpp degradative pathway, involving NudG and MutT, was uncovered. This report thus significantly expands the known cohort of (p)ppGpp targets in *E. coli*.

## IMPORTANCE

Antibiotic resistance and tolerance exhibited by pathogenic bacteria have resulted in a global public health crisis. Remarkably, almost all bacterial pathogens require the alarmone (p)ppGpp to be virulent. Thus, (p)ppGpp not only induces tolerance of nutritional limitations and chemical insults, including antibiotics, but is also often required for induction of virulence genes. However, understanding of the molecular targets of (p)ppGpp and the mechanisms by which (p)ppGpp influences bacterial physiology is incomplete. In this study, a systematic approach was used to uncover novel targets of (p)ppGpp in *E. coli*, the best-studied model bacterium. Comprehensive comparative studies of the targets revealed conserved target pathways of (p)ppGpp in both Gram-positive and -negative bacteria and novel targets of (p)ppGpp, including an alternative degradative pathway of (p)ppGpp. Thus, our discoveries may help in understanding of how (p)ppGpp increases the stress resilience and multidrug tolerance not only of the model organism *E. coli* but also of the pathogenic organisms in which these targets are conserved.

## INTRODUCTION

Magic spots, namely, guanosine penta- and tetraphosphate molecules, collectively known as (p)ppGpp, are universal signaling molecules in bacteria and plastids (1), wherein they play significant roles in various stress responses, including tolerance of antibiotics (2), and biofilm formation and virulence gene expression (3). Initially, (p)ppGpp were discovered as signaling molecules whose syntheses were strongly induced by amino acid limitation and thereby defined the stringent response (4). Later analyses showed that (p)ppGpp levels were increased by many other stress conditions, including conditions met by almost all bacterial pathogens in their host organisms (3). Thus, (p)ppGpp is in general required for bacteria to survive under natural conditions (5). Remarkably, the identification of (p)ppGpp binding proteins in *Escherichia coli* K-12, the best-studied model organism, has not been systematically pursued.

Confronted with various environmental insults, bacteria adjust their physiology globally primarily via the action of (p)ppGpp, which reprograms cellular metabolism from rapid growth to slow growth or to dormancy (6, 7). In the Gram-negative bacterium *E. coli*, (p)ppGpp binds to two sites of RNA polymerase (RNAP) (8). Together with the transcriptional regulator DksA, (p)ppGpp tunes the ability of RNAP to preferentially recognize promoters of the genes involved in counteracting stresses (9–13). Analysis of *E. coli* revealed that (p)ppGpp levels dramatically increased 10- to 100-fold during shifts to amino acid starvation (4, 14–16). This response depends on the presence of RelA [(p)ppGpp synthetase I], which is active when bound to the ribosomal A-site together with cognate, uncharged tRNA (17–19). (p)ppGpp reprograms RNAP to actively transcribe genes involved in biosynthesis of amino acids, while transcription of genes encoding ribosome proteins, rRNA, and tRNA is repressed. These dramatic physiological changes reduce the cell growth rate or induce dormancy (9–11, 13, 20, 21). Via such transcriptional reprogramming, (p)ppGpp affects the expression of between 500 and 1,400 genes (15, 21).

Besides the profound effects on the global transcription pattern, (p)ppGpp also influences (directly or indirectly) many other cellular processes in *E. coli*, such as DNA replication, translation, and metabolism (7, 22). (p)ppGpp has weak inhibitory effects on DnaG from *E. coli*, with half maximal inhibitory concentration (IC_50_) values in the low millimolar range (23). In *E. coli*, (p)ppGpp also binds directly to translation initiation factor 2 (InfB, IF2) (24), elongation factor G (EF-G, FusA) (25), BipA (26), ribosome release factor 3 (RF3, PrfC) (27), and the essential GTPase ObgE (28), probably inhibiting target functions in all cases. Various metabolic enzymes were also found to bind (p)ppGpp in *E. coli*. First, the purine salvage pathway enzymes Gpt, Hpt, and Apt as well as GuaB and PurA were reported to be inhibited by (p)ppGpp (29–32). Second, LdcI, the inducible lysine decarboxylase involved in counteracting acid stress, was serendipitously found to cocrystalize with ppGpp and ppGpp was proposed to allosterically regulate the activity of LdcI (33). Furthermore, additional three decarboxylases, LdcC, SpeF, and SpeC, that are involved in polyamine synthesis also bind ppGpp (34). In addition, (p)ppGpp stimulates the accumulation of inorganic polyphosphate by specifically inhibiting the polyphosphate hydrolase PPX in a competitive manner (inhibitory constant *K*_*i*_ = 10 and 200 µM for pppGpp and ppGpp, respectively) (35). Finally, ppGpp allosterically stimulates the activity of RelA in *E. coli* (36).

In this study, we used the differential radial capillary action of ligand assay (DRaCALA) to systematically identify novel (p)ppGpp binding proteins of *E. coli* K-12. We discovered new (p)ppGpp binding proteins involved in nucleotide homeostasis (YgdH), ribosome biogenesis and translational processes (RsgA, Era, HflX, and LepA), and maturation of dehydrogenases (HypB) and a host of novel proteins that can metabolize (p)ppGpp (MutT, NudG, TrmE, NadR, PhoA, and UshA). The use of DRaCALA also allowed us to comparatively analyze previously reported and newly identified (p)ppGpp targets. Our *in vivo* and *in vitro* studies raise the possibility of the existence of an alternative pathway for degradation of (p)ppGpp in *E. coli*. This work thus significantly expands the broad range of (p)ppGpp targets in the well-studied model organism *E. coli* K-12.

## RESULTS AND DISCUSSION

### Systematic identification of (p)ppGpp binding proteins of *E. coli* K-12.

To identify novel (p)ppGpp binding proteins, we used DRaCALA, a recently developed technique that allows fast detection of small molecule–protein interactions (37–39). DRaCALA exploits the differential diffusion rates of free and protein-bound radiolabeled small ligands on a nitrocellulose membrane (37). Despite the known issues (40), DRaCALA remains one of the most powerful tools devised so far for identifying small ligand binding proteins in a systematic manner. Previously, novel targets of bacterial signaling nucleotides, such as c-di-GMP (cyclic diguanylate monophosphate), c-di-AMP, cAMP, and, recently, also (p)ppGpp (in *Staphylococcus aureus*), had successfully been identified by DRaCALA (37–41). To employ DRaCALA, we used the ASKA plasmid library consisting of a complete set of *E. coli* K-12 genes encoding N-terminally His-tagged proteins encoded by a high-copy-number plasmid (42). The ASKA library strains were grown in microtiter plates, plasmid-borne genes were induced by IPTG (isopropyl-β-d-thiogalactopyranoside), and cell lysates were prepared as described in Materials and Methods. Next, radiolabeled pentaphosphate [α-^32^P]pppGpp was synthesized from [α-^32^P]GTP (PerkinElmer) and ATP, using a C-terminally truncated form of the *Streptococcus equisimilis* enzyme Rel_Seq(1−385)_ as previously described (43). Over 94% of [α-^32^P]GTP was converted to [α-^32^P]pppGpp as assessed by thin-layer chromatography (TLC), and the tetraphosphate [α-^32^P]ppGpp was then synthesized from [α-^32^P]pppGpp by the use of *E. coli* protein GppA (more than 92% conversion; see Fig. S1A in the supplemental material). To simplify the screening and to identify proteins binding pppGpp or ppGpp or both, equal amounts of [α-^32^P]pppGpp and [α-^32^P]ppGpp were mixed and a proteome-wide DRaCALA screen was subsequently performed as described previously (38, 39). Via this screening, lysates of 21 ASKA collection strains in total were found to contain proteins that bound (p)ppGpp (RF3 [twice], MutT, Gpt, PhoA, UshA, NudG, YgdH, Era, HypB, IF2, TrmE, NadR, Hpt, Der, RsgA, LepA, ObgE, HflX, EF-Tu, and RelA). The presence of (p)ppGpp binding proteins in these lysates and their binding of (p)ppGpp were confirmed (Fig. S1B to D). Subsequently, gene identities were confirmed by sequencing of the ASKA plasmids. As described in more detail below, eight proteins (Gpt, Hpt, IF2, Der, RF3, ObgE, EF-Tu, and RelA) were previously known to bind (p)ppGpp whereas the other 12 proteins represent newly identified targets of (p)ppGpp. Eleven proteins previously reported to bind (p)ppGpp (LdcC, LdcI, SpeF, SpeC, GuaB, PPX, PurA, Apt, DnaG, BipA, and GppA) were not identified in this screening. This could have been due to their poor expression, poor solubility, the presence of extra residues at both ends of the proteins, or their relative low binding affinities to (p)ppGpp as discussed below for LdcI, Apt, PurA, and DnaG. Of note, some *E. coli* proteins could not be expressed from the ASKA library strains in soluble forms (42), representing a subpool of proteins that may contain more (p)ppGpp binding targets. Nevertheless, we identified here approximately two-thirds (20/31) of all currently known (p)ppGpp binding proteins and revealed one-third more (12/31) new targets (Table 1; see also Table S1 in the supplemental material). Based on the physiological functions, the 12 new (p)ppGpp binding proteins were binned into four major groups involved in (i) nucleotide metabolism, (ii) ribosome biogenesis and translation, (iii) maturation of dehydrogenases, and (iv) metabolism of (p)ppGpp. In the following, we describe a comprehensive and yet preliminary comparative analysis of these 12 novel (p)ppGpp binding proteins, together with other relevant known targets of (p)ppGpp in *E. coli* K-12, in order to provide a global view of (p)ppGpp binding proteins in *E. coli*.

10.1128/mBio.02188-17.1 FIG S1  Analysis of hits identified by screening of the ASKA strain collection using DRaCALA. (A) TLC assessment of synthesized [α-^32^P]pppGpp and [α-^32^P]ppGpp used for DRaCALA screening. A minimum conversion ratio of 92% was determined for conversion of [α-^32^P]GTP to [α-^32^P]pppGpp and conversion of [α-^32^P]pppGpp to [α-^32^P]ppGpp. (B) Coomassie-stained 4% to 12% NuPAGE gel of *E. coli* lysates containing each target protein either without or with IPTG induction. Expected proteins of the correct sizes are indicated by arrows. (C) Representative DRaCALA spots of each cell lysate binding [α-^32^P](p)ppGpp (2 nM) determined as described for panel B. (D) Quantification of fractions bound as described for panel C. Download FIG S1, TIF file, 1.9 MB. Copyright © 2018 Zhang et al. 2018 Zhang et al. This content is distributed under the terms of the Creative Commons Attribution 4.0 International license.

10.1128/mBio.02188-17.8 TABLE S1  List of proteins that bind or cleave (p)ppGpp. Download TABLE S1, DOCX file, 0.1 MB. Copyright © 2018 Zhang et al. 2018 Zhang et al. This content is distributed under the terms of the Creative Commons Attribution 4.0 International license.

**TABLE 1:** (p)ppGpp binding proteins in *E. coli* and their presence in other bacteria^a^

Protein(s)	Source or species or reference(s)	
	*E. coli*	Other bacteria
Purine nucleotide biosynthesis		
* YgdH*	This study	
Gpt	This study (29, 30)	
Hpt	This study (29, 30)	*B. subtilis* (46); *S. aureus* (39)
**GuaB**	31	*B. subtilis* (46)
**PurA**	31	
**Apt**	30	
		
Ribosome and translation		
*LepA*	This study	
*Era*	This study	*S. aureus* (39)
*HflX*	This study	*S. aureus* (39)
*RsgA*	This study	*S. aureus* (39)
Der	This study (49)	SaDer; *S. aureus* (this study)
RF3(PrfC)	This study (27)	*D. vulgaris* (27)
ObgE	This study (28)	*B. subtilis* (50)
EF-Tu	This study (71)	
EF-G	This study (71)	
IF2(InfB)	This study (24)	
**BipA**	26	
		
DNA replication		
**DnaG**	23	*B. subtilis* (72)
		
Transcription		
**RNAP/DksA**	8	
		
(p)ppGpp homeostasis		
*MutT*	This study	Ndx8 (*T. thermophilus*) (64)
*NudG*	This study	
*TrmE*	This study	
*NadR*	This study	
*PhoA*	This study	
*UshA*	This study	
RelA	This study (36)	
**SpoT**		
**GppA**	51	
		
Metabolism		
*HypB*	This study	
**LdcI**	33	
**LdcC, SpeC, SpeF**	34	
**PPX**	35	

a ppGpp binding proteins identified prior to and not in this study are indicated in boldface; (p)ppGpp binding proteins newly identified here are indicated in italic.

### The purine nucleotide salvage biosynthesis pathways are conserved targets of (p)ppGpp in both Gram-positive and -negative bacteria.

**FIG 1:**
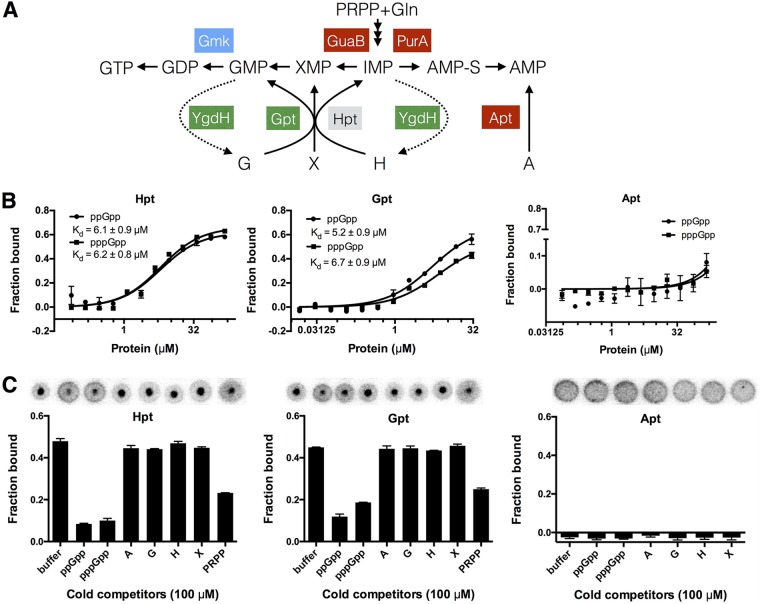
GTP biosynthesis and salvage pathways are targeted by (p)ppGpp. (A) Schematic of purine biosynthesis pathways with (p)ppGpp targets highlighted by colored boxes. Green indicates *E. coli* targets identified here; blue indicates specific *Bacillus*/*Staphylococcus* targets; red indicates *E. coli* targets reported previously but not confirmed in this study; gray indicates a target found in *E. coli*, *Bacillus*, and *Staphylococcus*. G, guanine; X, xanthine; H, hypoxanthine; A, adenine; PRPP, phosphoribosyl pyrophosphate; Gln, glutamine. (B) Binding curves and apparent *K*_*d*_ values for *E. coli* Gpt, Hpt, and Apt binding pppGpp and ppGpp (2 nM [each]). The average values for bound fractions and standard errors of the means (SEM) determined for at least three replicates were plotted and the curve-fitted and *K*_*d*_ values determined as previously described (37). The apparent *K*_*d*_ values corresponding to each protein-ligand interaction are shown. (C) Competition assay of Gpt, Hpt, and Apt (20 μM [each]) binding [α-^32^P]ppGpp (2 nM) in the presence of cold competitors (100 μM). The average values for bound fractions and standard errors of the means (SEM) determined for at least three replicates were plotted. Representative DRaCALA spots are shown above the respective diagrams.

In this study, YgdH, a recently established nucleotide nucleosidase (44), was identified as a new target of (p)ppGpp. To understand more comprehensively the role of (p)ppGpp in purine/pyrimidine metabolism, we first used DRaCALA to reinvestigate other reported (p)ppGpp binding proteins involved in this pathway. *E. coli* synthesizes purine nucleotides through both *de novo* and salvage pathways, with the former using phosphoribosyl pyrophosphate (PRPP) and glutamine, and the latter using PRPP and nucleobases (Fig. 1A). All three purine phosphoribosyltransferases in the salvage pathway (Gpt, Hpt, and Apt) were shown before to be inhibited by ppGpp with different affinities (29–32). ppGpp seems to have stronger inhibitory effect on Gpt/Hpt than on Apt (IC_50_, ca. 85 µM for Gpt/Hpt versus 1.5 mM for Apt). Considering the intracellular concentrations of (p)ppGpp in stressed *E. coli* cells (1 to 2 mM) (16, 45), Apt may not be a major physiological target of (p)ppGpp. Consistently, this study identified both Gpt and Hpt, but not Apt, as (p)ppGpp binding proteins (Fig. 1B and C; see also Fig. S1B to D). The *E. coli* Gpt, Hpt, and Apt proteins from the ASKA collection were purified to homogeneity (Fig. S2), and apparent disassociation constant (*K*_*d*_) values were determined for their binding of ppGpp and pppGpp as explained in Materials and Methods. Gpt and Hpt have high and comparable levels of binding affinity for both ppGpp and pppGpp (*K*_*d*_ = 5.2 ± 0.9 µM and 6.7 ± 0.9 µM for Gpt binding ppGpp and pppGpp, respectively; *K*_*d*_ = 6.1 ± 0.9 µM and 6.2 ± 0.8 µM for Hpt binding ppGpp and pppGpp, respectively). However, the apparent *K*_*d*_ for Apt binding (p)ppGpp could not be determined by DRaCALA and no significant binding of (p)ppGpp could be observed even when 150 µM Apt was used (Fig. 1B and C). This indicates that Apt probably is not as strongly affected by (p)ppGpp as Gpt/Hpt. Similarly to *E. coli*, *S. aureus* and *Bacillus subtilis* have a HprT that is strongly inhibited by (p)ppGpp (IC_50_ = 11 µM ppGpp for *B. subtilis* HprT and *K*_*d*_ = 0.37 to 0.75 µM for *S. aureus* HprT) (39, 46). Of note, *B. subtilis* (as well as *S. aureus*) has only HprT, which is more closely related to *E. coli* Hpt (51% amino acid identity, 95% coverage, E value of 9e−56) than to Gpt (29% amino acid identity, 76% coverage, E value of 2e−05). *E. coli* Gpt and Hpt are potentially paralogous proteins (29% amino acid identity, 64% coverage, E value of 5e−11), and they have overlapping substrates, with Hpt favoring hypoxanthine over guanine and Gpt favoring xanthine/guanine over hypoxanthine.

10.1128/mBio.02188-17.2 FIG S2  Coomassie-stained 4% to 12% NuPAGE gels of purified (p)ppGpp target proteins investigated in this study. Download FIG S2, TIF file, 0.8 MB. Copyright © 2018 Zhang et al. 2018 Zhang et al. This content is distributed under the terms of the Creative Commons Attribution 4.0 International license.

ppGpp was shown to competitively inhibit the enzymatic activities of Gpt and Hpt of *E. coli* (29). However, it remains unclear whether the inhibitory effect of (p)ppGpp on Gpt/Hpt is competitive with respect to either PRPP or the purine nucleobases (30). DRaCALA is known to be a useful tool for deciphering the binding mechanisms (37). To investigate this, we performed a competition assay where high concentrations (100 µM) of cold nucleobases (guanine, xanthine, hypoxanthine, and adenine) and PRPP were used to compete for the bound hot [α-^32^P]ppGpp on purified Gpt and Hpt proteins. Cold (unlabeled) (p)ppGpp and binding buffer were used as controls. (p)ppGpp and PRPP were able to partially outcompete the bound radiolabeled [α-^32^P]ppGpp; however, none of the purine nucleobases displayed this capacity (Fig. 1C), indicating that (p)ppGpp binds at the pocket occupied by PRPP on both Gpt and Hpt. This is understandable, considering the fact that both Gpt and Hpt use PRPP as a common substrate but prefer different nucleobases.

Besides the salvage pathway, both GuaB and PurA in the *de novo* pathway of ATP/GTP biosynthesis were shown to be inhibited by ppGpp in *E. coli* (29, 31, 47). The inhibitory constant (*K*_*i*_) values were reported to be between 30 and 50 µM for GuaB and between 50 to 140 µM for PurA. However, our screening failed to identify either PurA or GuaB as a (p)ppGpp binding protein. To study this discrepancy further, we tried to purify both proteins; however, GuaB could not be purified to homogeneity in large amounts. Inspection of the GuaB tetramer structure from *Vibrio cholerae* (PDB 4FXS) suggests that the extra N- and C-terminal residues on the pCA24N vector may affect protein folding and solubility and thus binding of (p)ppGpp. This probably explains why GuaB was not identified in our screening. A similar scenario may apply to other known (p)ppGpp binding proteins that were not identified in this study, such as LdcI (data not shown). In contrast, PurA was successfully purified and used for measurement of its binding affinities to (p)ppGpp; however, no specific binding could be observed even when PurA was used at up to 150 µM (Fig. S3). Inspection of the PurA complex structure with ppG2′3′p and IMP (PDB 1CH8) and with GDP and IMP (PDB 1CIB) (32) indicated that the extra N- and C-terminal residues are unlikely to affect protein structure (data not shown) and thus the ability to bind (p)ppGpp. Instead, this may reflect the same scenario as discussed above for Apt (PDB 2DY0). Of note, DRaCALA was known to primarily detect strong protein-ligand interactions and this may explain the lack of signal corresponding to ppGpp binding of Apt, PurA, and also DnaG, which all displayed high IC_50_s (ranging from 100 µM to mM) for ppGpp (23, 29, 30). Of note, GuaB from *B. subtilis* was also found to be not significantly inhibited by ppGpp (IC_50_, ca. 0.3 to 0.5 mM) (46).

10.1128/mBio.02188-17.3 FIG S3  (p)ppGpp does not significantly bind to purified PurA. (A) Competition assay of PurA (20 μM) binding α-^32^P-labeled ppGpp and pppGpp (2 nM [each]) in the presence of cold competitors (100 μM). (B) Binding curves and *K*_*d*_ determinations for PurA binding α-^32^P-labeled ppGpp and pppGpp (2 nM [each]). Download FIG S3, TIF file, 0.8 MB. Copyright © 2018 Zhang et al. 2018 Zhang et al. This content is distributed under the terms of the Creative Commons Attribution 4.0 International license.

Together with data published by other groups (39, 46), our observations confirm that the salvage pathway of GTP biosynthesis (Gpt/Hpt) is a highly conserved target of (p)ppGpp in both Gram-positive and -negative bacteria.

### YgdH, a protein involved in nucleotide metabolism, binds (p)ppGpp.

With better understanding of the known (p)ppGpp targets in the purine biosynthesis pathway, we next studied YgdH (ECK2790), which we identified as a novel (p)ppGpp binding protein (Fig. S4A). YgdH was recently found through high-throughput mass spectrometry (Mass-Spec) studies to degrade nucleotide 5′-monophosphate (including AMP, IMP, GMP, CMP, and UMP) into ribose phosphate and nucleobases, possibly playing a role in purine/pyrimidine salvage pathways (44). Competition assay results showed that the binding of (p)ppGpp to YgdH was specific, because only excess levels of cold competitor (p)ppGpp (100 µM) but not of GTP/GDP or ATP/ADP could outcompete (p)ppGpp (data not shown).

10.1128/mBio.02188-17.4 FIG S4  YgdH binds (p)ppGpp. (A) Original DRaCALA screening plate that identified YgdH (indicated by red circle). (B) FPLC profiles of purified His_6_-YgdH (53 kDa) (left) and protein standards (right) indicate that His_6_-YgdH forms a tetramer. Download FIG S4, TIF file, 1 MB. Copyright © 2018 Zhang et al. 2018 Zhang et al. This content is distributed under the terms of the Creative Commons Attribution 4.0 International license.

**FIG 2:**
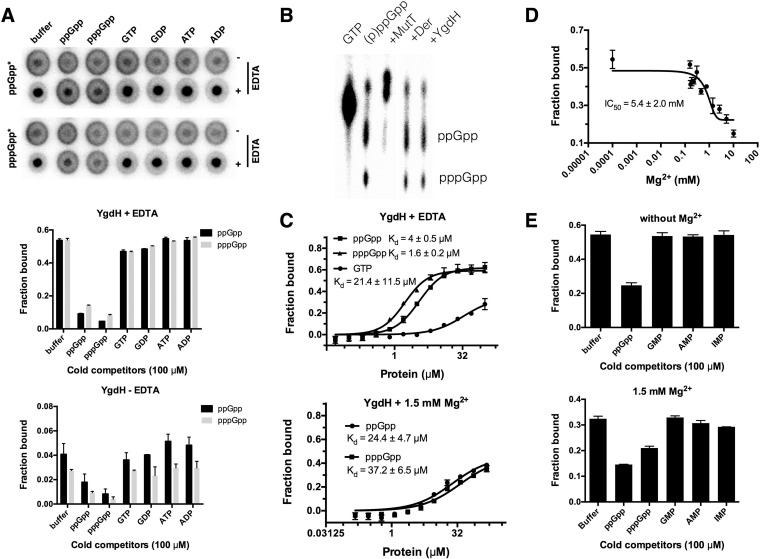
YgdH binds (p)ppGpp antagonistically with magnesium. (A) Competition assay of purified YgdH protein (20 μM) binding a 1:1 mixture of ppGpp and pppGpp (2 nM [each]) in the absence or presence of EDTA. Representative DRaCALA spots and quantifications (average values for bound fractions and standards errors of the means [SEM]) of binding signals are shown. (B) Thin-layer chromatography (TLC) of DRaCALA binding reactions determined by using 1.5 M K_2_HPO_4_ (pH 3.4) as the mobile phase. Binding reactions performed with purified MutT, Der, or YgdH were run in parallel with standards of [α-^32^P]GTP and a mixture of [α-^32^P]ppGpp and [α-^32^P]pppGpp (2 nM [each]). (C) Binding curves and *K*_*d*_ determinations for YgdH interacting with α-^32^P-labeled ppGpp, pppGpp, and GTP (2 nM [each]) without or with 1.5 mM MgCl_2_. The apparent *K*_*d*_ values corresponding to each protein-ligand interaction are shown. (D) Magnesium (0 to 10.15 mM) IC_50_ determinations of binding of [α-^32^P]ppGpp (2 nM) to YgdH (50 μM). IC_50_ values are shown. (E) Competition assay of YgdH (50 μM) binding [α-^32^P]ppGpp (2 nM) in the presence of 100 μM cold competitors [including (p)ppGpp and the substrates of YgdH (GMP, AMP, and IMP)] without or with 1.5 mM magnesium.

YgdH was subsequently purified to homogeneity, and a tetramer of YgdH was observed from the size exclusion fast protein liquid chromatography (FPLC) profile, consistent with crystal data of YgdH homologues (such as YgdH in *Vibrio* PDB 4NPA; Fig. S4B). Surprisingly, purified YgdH protein (20 μM) was not able to significantly bind (p)ppGpp (Fig. 2A), raising the possibility that purified YgdH degrades (p)ppGpp. To test this, the binding reactions were analyzed by TLC. However, (p)ppGpp was not degraded by YgdH, and positive (MutT) and negative (Der) controls were included for specificity (Fig. 2B). Instead, we thought it possible that a component in the binding buffer (containing 40 mM Tris [pH 7.5], 100 mM NaCl, 10 mM MgCl_2_) prevented the binding of (p)ppGpp to YgdH. We therefore tested whether magnesium inhibited the binding of (p)ppGpp by adding excess (2.5×) EDTA (25 mM final concentration). Indeed, addition of EDTA restored the strong binding of (p)ppGpp to YgdH (increasing it by more than 10-fold; Fig. 2A). A competition assay with purified YgdH showed that only cold (p)ppGpp effectively outcompeted hot (p)ppGpp in both the presence and absence of EDTA, confirming the specificity of the interaction (Fig. 2A). The binding affinities (*K*_*d*_) of YgdH to ppGpp and pppGpp were determined by DRaCALA to be 4 ± 0.5 µM and 1.6 ± 0.2 µM, respectively, in the presence of EDTA (and thus in the absence of magnesium), which were values much lower than those corresponding to its affinity for GTP (*K*_*d*_ = 21.4 ± 11.5 µM) (Fig. 2C).

Since magnesium is the second most abundant of the metal ions present in most bacteria, the inhibitory effect of 10 mM magnesium on binding of (p)ppGpp to YgdH seems to render the binding of no physiological relevance. To study this further, serial dilution of magnesium from 10.15 mM to 0 mM was used to determine its effect on (p)ppGpp binding to YgdH and to measure the IC_50_. An IC_50_ of 5.4 ± 2.0 mM was obtained (Fig. 2D), which was above the range concentrations of free cytoplasmic Mg^2+^ in *E. coli* (1 to 2 mM) as previously reported (48), arguing that the binding of (p)ppGpp to YgdH in *E. coli* could have physiological consequences. Furthermore, we tried to measure the binding affinities of YgdH in the presence of increasing concentrations of magnesium, and consistently significant levels of binding of ppGpp were found in the presence of 1.5 mM but not in the presence of 3, 5, or 10 mM Mg^2+^. The apparent *K*_*d*_ values were thus determined to be 24.4 ± 4.7 µM for ppGpp and 37.2 ± 6.5 µM for pppGpp with 1.5 mM Mg^2+^ present (Fig. 2C). The decreased binding affinities to both ppGpp and pppGpp by YgdH in the presence of magnesium argue that, under conditions where intracellular magnesium levels drop, (p)ppGpp could more effectively bind and regulate the function of YgdH.

The magnesium-dependent inhibition of (p)ppGpp binding to YgdH is unique. First, it is different from the binding of (p)ppGpp by MutT, NudG, and NadR (see below), proteins that require magnesium to bind and cleave (p)ppGpp. Instead, the binding of (p)ppGpp by YgdH is similar to that seen with the genuine (p)ppGpp binding proteins, such as Der and RF3 (see below), which represent cases in which magnesium is not required for (p)ppGpp binding. From this perspective, the binding of (p)ppGpp by YgdH probably has some physiological significance, especially under conditions of low levels of intracellular magnesium. To study further if (p)ppGpp binds at the substrate binding site of YgdH, a competition assay was done with the reported YgdH substrates GMP, AMP, and IMP. However, none of them could outcompete ppGpp whether magnesium was absent or present at 1.5 mM (Fig. 2E), indicating that ppGpp binds at a site different from the substrate binding pocket and allosterically regulates the function of YgdH. Taking the results together, the inverse relationship between magnesium concentration and binding affinity of (p)ppGpp to YgdH suggests a potential link between intracellular magnesium homeostasis and the regulation of YgdH function by (p)ppGpp in *E. coli*. Further studies are ongoing to decipher the underlying molecular mechanism.

### Many highly conserved, translational GTPases bind (p)ppGpp.

**FIG 3:**
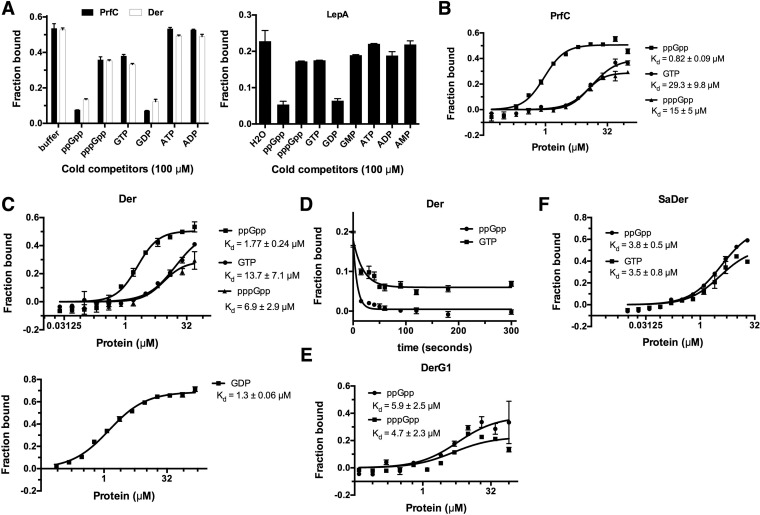
Translational GTPases are conserved targets of (p)ppGpp. (A) Competition assay of RF3 and Der (20 μM) and LepA (10 μM) binding [α-^32^P]ppGpp (2 nM) in the presence of cold competitors (100 μM). (B and C) Binding curves and *K*_*d*_ determination of RF3 (B) and Der (C) binding of α-^32^P-labeled ppGpp, pppGpp, GTP, or GDP (2 nM [each]). At least three replicates were performed. The apparent *K*_*d*_ values corresponding to each protein-ligand interaction are shown. (D) Dissociation curves for Der (50 μM) and [α-^32^P]ppGpp (2 nM) in the presence of either ppGpp or GTP (100 μM) (cold). (E and F) Binding curves and *K*_*d*_ determination for DerG1 (E) and SaDer (F) binding α-^32^P-labeled ppGpp, pppGpp, or GTP (2 nM [each]). At least three replicates were performed. The apparent *K*_*d*_ values are shown for each protein-ligand interaction.

A number of highly conserved GTPases involved in translation or ribosome biogenesis are known to bind (p)ppGpp, including initiation factor 2 (IF2), EF-G, release factor 3 (RF3), Der, ObgE, and BipA (24–27, 39, 49, 50). All of these proteins except BipA were identified as (p)ppGpp binding proteins in this study (Fig. S1). Here, we identified 4 novel GTPases, HflX, RsgA, Era, and LepA, as new (p)ppGpp binding proteins in *E. coli* (Fig. S1 and S5). To obtain a more comprehensive understanding of how (p)ppGpp affects the newly identified and previously known GTPases, we attempted to purify them (Fig. S2). Five GTPases (Der, RF3, ObgE, Era, and LepA) were obtained in sufficient amounts for detailed biochemical analysis. The binding specificities of these proteins to (p)ppGpp were confirmed using the competition assay described above (Fig. 3A; see also Fig. S5A and B), and GTP/GDP could outcompete the bound (p)ppGpp to some extent, indicating competitive binding of (p)ppGpp at the GTP/GDP binding pockets of the GTPases. In addition, ppGpp and GDP seem to have higher affinities than pppGpp and GTP for all five proteins as indicated by the lower values for the fraction of bound hot ppGpp seen when cold ppGpp and GDP were present (Fig. 3A; see also Fig. S5A). Similar phenomena were observed for HflX, EF-G, and IF2 (Fig. S5A). In accordance with this, when the apparent *K*_*d*_ values of (p)ppGpp binding to these five proteins were determined by DRaCALA using serially diluted proteins, the binding affinity to ppGpp was observed to be higher than that to pppGpp for all of them (Fig. 3B and C; see also Fig. S5E). Of note, although the apparent *K*_*d*_ of LepA to (p)ppGpp could not be determined due to the low concentration of soluble proteins achieved, tighter binding of ppGpp than pppGpp could be observed from the binding curves as well (Fig. S5E). The apparent *K*_*d*_ values of Der binding to both GTP and GDP were also determined, with GDP having *K*_*d*_ values similar to those determined for ppGpp (1.3 ± 0.1 µM versus 1.8 ± 0.2 µM) and GTP having *K*_*d*_ values similar to those determined for pppGpp (13.7 ± 7.1 µM versus 6.9 ± 2.9 µM). In addition, the binding affinity of RF3 to GTP was determined to be 29.3 ± 9.8 µM, a level similar to its binding of pppGpp (*K*_*d*_ = 15 ± 5 µM) but much lower than that seen with ppGpp (*K*_*d*_ = 0.82 ± 0.09 µM). The observed relatively higher binding affinities to ppGpp and GDP than to pppGpp and GTP prompted us to probe further the binding kinetics. For this, the fraction of bound [α-^32^P]GTP on Der was followed after chase experiments performed using an excess concentration (100 µM) of either cold GTP or cold ppGpp. It was observed that the chance of [α-^32^P]GTP rebinding on Der decreased much faster when cold ppGpp was applied than when cold GTP was applied (Fig. 3D). A similar phenomenon was observed for RF3 when radiolabeled ppGpp was used (Fig. S5F). These data are thus consistent with the observation that both Der and RF3 have higher affinities to ppGpp and GDP than to pppGpp and GTP. Considering that ppGpp, instead of pppGpp, is the major species produced during the stringent response (16, 51), these data indicate that ppGpp is more potent than pppGpp in binding these GTPases. More importantly, GDP and GTP were found to behave like ppGpp and pppGpp, respectively, in competition assays and in *K*_*d*_ determinations (Fig. 3A to D; see also Fig. S5A, B, E, and F). The 5′-end moieties of GDP and ppGpp and of GTP and pppGpp are the same. The concentration of ppGpp is known to rise even higher than that of GDP under stressful conditions (GDP is also consumed to make ppGpp). Taken together, these data indicate that, under stressful conditions, it is mainly ppGpp that competitively binds the translational GTPases and drives their equilibria away from the active GTP-bound active states, to slow translation and cell growth.

10.1128/mBio.02188-17.5 FIG S5  Ribosome-associated GTPases are conserved targets of (p)ppGpp. (A) Competition assay of purified HflX (6.5 μM), EF-G (11 μM), IF2 (2.8 μM), Era (20 μM), and ObgE (20 μM) binding [α-^32^P]ppGpp (2 nM) in the presence of cold competitors (100 μM). (B) Competition assay of purified RF3 and Der (20 μM) binding [α-^32^P]ppGpp and [α-^32^P]pppGpp (2 nM [each]) in the presence of cold competitors (100 μM). (C) Competition assay of purified DerG2KH (3 μM) and DerG1 (20 μM) binding [α-^32^P]ppGpp (2 nM) in the presence of cold competitors (100 μM). (D) Competition assay of purified SaRF3 (18.7 μM) and SaDer (12.1 μM) binding [α-^32^P]ppGpp (2 nM) in the presence of cold competitors (100 μM). At least two replicates were done. (E) Binding curves and *K*_*d*_ determination for purified Era, ObgE, and LepA binding α-^32^P-labeled ppGpp and pppGpp (2 nM [each]). At least two biological replicates were performed. The apparent *K*_*d*_ values corresponding to each protein-ligand interaction are shown. *K*_*d*_ values could not be determined for LepA binding (p)ppGpp. (F) Dissociation curves for Der (50 μM) and [α-^32^P]ppGpp (2 nM) in the presence of cold ppGpp, pppGpp, GTP, and GDP (100 μM [each]). Two biological replicates were made. (G) Schematic drawing of *E. coli* Der domains (G1, G2, and KH) and different constructs studied here (G1 and G2KH). Red bars indicate six-histidine tags. See Materials and Methods for details. Download FIG S5, TIF file, 2 MB. Copyright © 2018 Zhang et al. 2018 Zhang et al. This content is distributed under the terms of the Creative Commons Attribution 4.0 International license.

Interestingly, the ribosome-associated GTPases (HflX, RsgA, Era, and RbgA) from *S. aureus* were recently found to bind (p)ppGpp (39). Inspection of the reported binding affinities to (p)ppGpp and GTP by these GTPases in *S. aureus* revealed the same pattern, with higher affinities of binding of these GTPases to ppGpp than to pppGpp and GTP (see Table S1 in reference 39). Taken together, these data argue for a conserved mode of action of ppGpp in bacteria of different phylogenies, where ppGpp produced from GDP and pppGpp (synthesized by consuming GTP) in cells under stressful conditions quickly (because the conditions are kinetically favorable for binding), competitively, and, more importantly, reversibly replaces and prevents the rebinding of GTP.

In an attempt to understand the binding of ppGpp in preference to pppGpp, we studied Der, a unique GTPase with two consecutive GTP binding domains (G domains) fused with a C-terminal RNA binding KH domain. Previous studies showed that binding of either G domain to GTP or GDP regulates the ability of Der to interact with ribosome subunits (52). Furthermore, the ribosome-bound conformation of *E. coli* Der (PDB 3J8G) is significantly different from the free form of Der of *Thermotoga maritima* (PDB 1MKY) in that the second G domain (G2) and the KH domain make a dramatic rotation with respect to the first G domain (G1) upon ribosome binding. A long, flexible linker (residues A167 to P202 [*E. coli* Der protein numbering]) between the two G domains is important for mediating the conformational change. To study the ppGpp binding property of both G domains, the segments containing G1 (residues 2 to 184) or G2 plus KH (G2KH; residues 185 to 490) were separately cloned as C-terminal and N-terminal histidine-tagged proteins, respectively, by adding a histidine tag adjacent to the flexible linker to minimize potential adverse effects of the histidine tag (Fig. S5G). However, G2KH turned out to be unstable whereas G1 was more stable and readily purified. As shown in Fig. S5C, unlike full-length Der, G1 appears to bind ppGpp and GDP in preference to pppGpp and GTP in competition assays. However, similar binding affinities to ppGpp and pppGpp (*K*_*d*_ = 5.9 ± 2.5 µM and *K*_*d*_ = 4.7 ± 2.3 µM, respectively) were determined for G1 (Fig. 3E). These data thus suggest a cross communication between G1 and G2KH domains of Der that is essential for its preferential binding of ppGpp over pppGpp.

The ribosome and its associated GTPases are highly conserved across different bacterial phylogenies. To test whether homologues of Der and RF3 in *Firmicutes* also bind (p)ppGpp, we cloned the corresponding genes from *S. aureus* strain Newman (NWMN_1384 and NWMN_0890, respectively) expressing N-terminal histidine-tagged proteins (denoted *S. aureus* RF3 [SaRF3] and SaDer). SaRF3 was not very soluble and showed very weak binding (if any) of ppGpp when a 12 µM concentration of protein was used (Fig. S5D). SaDer seemed to bind ppGpp and GDP slightly better than pppGpp and GTP from the competition assay, but comparable *K*_*d*_ values were observed for ppGpp and GTP (*K*_*d*_ = 3.8 ± 0.5 µM for ppGpp versus *K*_*d*_ = 3.5 ± 0.8 µM for GTP) (Fig. 3F; see also Fig. S5D). Of note, the *K*_*d*_ values were higher than those seen with the other four GTPases studied in *S. aureus* (39) which were measured by DRaCALA as well. These data thus indicate that SaDer indeed binds ppGpp with physiological affinity but that it may not respond to increased levels of (p)ppGpp as strongly as other GTPases when *S. aureus* is stressed.

Taken together, these data uncovered new ribosome-related GTPases as (p)ppGpp targets in both *E. coli* (LepA, HflX, Era, and RsgA) and *S. aureus* (SaDer). More importantly, the conserved mode of action of ppGpp on ribosome-related GTPases was revealed for both Gram-positive and -negative strains, indicating that ribosome biogenesis and translational processes are highly conserved targets of (p)ppGpp in bacteria.

### HypB, a GTPase involved in maturation of dehydrogenases, binds (p)ppGpp.

**FIG 4:**
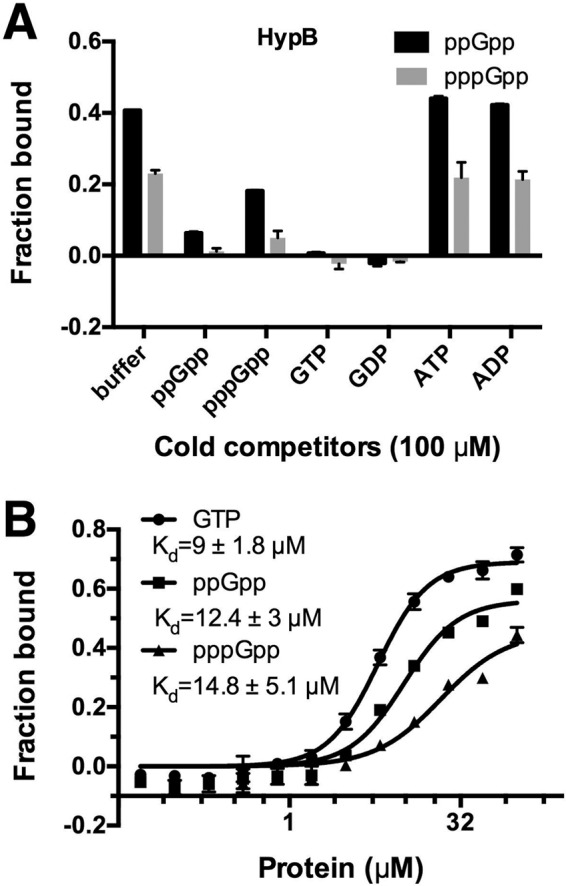
HypB specifically binds (p)ppGpp with physiological affinity. (A) Competition assay of HypB (20 μM) binding α-^32^P-labeled ppGpp and pppGpp (2 nM [each]) in the presence of cold competitors (100 μM). (B) Binding curves and *K*_*d*_ determination for HypB binding α-^32^P-labeled ppGpp, pppGpp, and GTP (2 nM [each]). Three replicates were performed, and the apparent *K*_*d*_ values are indicated.

HypB plays an essential role in conferring the nickel ion to, and therefore in the maturation of, all hydrogenase isoenzymes in *E. coli* (53, 54). Hydrogenases couple the oxidation of H_2_ to reduction of O_2_ and the conversion of formate to CO_2_ and H_2_, avoiding overacidification of the cytoplasm during fermentation (55), and increase fitness upon transition from anaerobic to aerobic conditions. HypB contains an N-terminal peptide involved in binding of Ni^2+^ and a C-terminal GTP binding domain. The GTP hydrolysis activity of HypB is essential for its function (56). Our competition experiment indicated that (p)ppGpp binds at the GTP binding pocket of HypB (Fig. 4A). Furthermore, the binding affinities of ppGpp and pppGpp for HypB (*K*_*d*_ = 12.4 ± 3 µM and 14.8 ± 5.1 µM, respectively) are comparable to that of GTP (*K*_*d*_ = 9.0 ± 1.8 µM) (Fig. 4B), raising the possibility that the function of HypB may be regulated by (p)ppGpp under certain redox conditions. As a facultative anaerobe, *E. coli* experiences a constant change of oxygen concentrations throughout the intestinal tract in host. Oxidative stress induced by H_2_O_2_ indeed triggers production of ppGpp in both *E. coli* and *Pseudomonas aeruginosa* (57, 58). The abrupt production of (p)ppGpp and its potential competitive inhibition of HypB may serve to halt the maturation of more hydrogenases, which would be unnecessary after *E. coli* adapted to more oxidative environments. However, the exact underlying molecular mechanism remains to be studied.

### Identification of new (p)ppGpp-hydrolyzing proteins.

The cellular level of (p)ppGpp is determined by its rates of synthesis and degradation. In beta- and gammaproteobacteria, including *E. coli*, two homologous proteins, RelA and SpoT, synthesize (p)ppGpp by transferring the terminal pyrophosphate of ATP onto the 3′-hydroxyl of the ribose ring of either GTP or GDP, producing pppGpp or ppGpp, respectively (6). In addition to the (p)ppGpp synthetic activity, SpoT possesses (p)ppGpp hydrolytic activity, whereas RelA has lost this activity. It is currently believed that SpoT cleaves off the 3′-pyrophosphate of (p)ppGpp and generates one molecule of GTP or GDP. Therefore, in *E. coli*, RelA and SpoT are responsible for the synthesis and degradation of (p)ppGpp. In the following, we describe the discovery of six novel (p)ppGpp-hydrolyzing proteins, MutT, NudG, TrmE, NadR, PhoA, and UshA.

**FIG 5:**
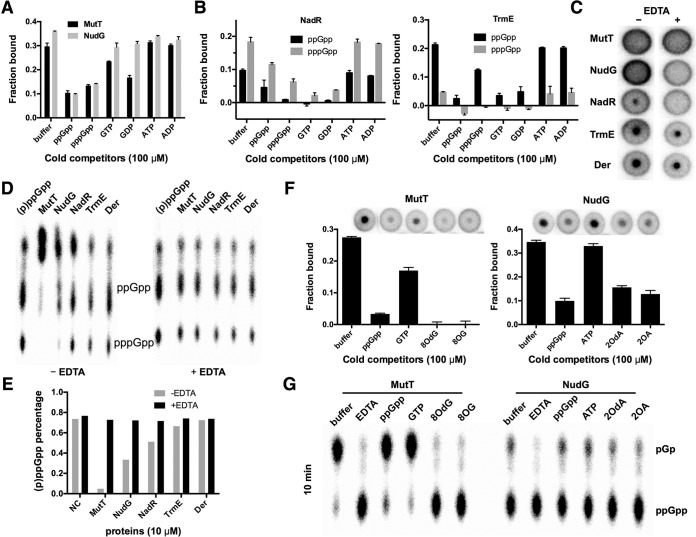
*In vitro* cleavage of ppGpp by MutT, NudG, NadR, and TrmE. (A) Competition assay of whole-cell lysates containing overexpressed MutT and NudG binding [α-^32^P]ppGpp (2 nM) in the presence of cold competitors (100 μM). (B) Competition assay of purified NadR (left) and TrmE (right) (20 μM [each]) binding α-^32^P-labeled ppGpp and pppGpp (2 nM [each]) in the presence of cold competitors (100 μM). (C) DRaCALA spots of purified proteins (10 μM) binding a mixture of α-^32^P-labeled ppGpp and pppGpp (2 nM [each]) in the absence or presence of EDTA (25 mM). (D) TLC assessment of cleavage products from the binding reactions described for panel C. A mixture of ppGpp and pppGpp was run as the standard, and both molecules are indicated. (E) Quantification of (p)ppGpp percentage determined as described for panel D. (F) Competition assay of whole-cell lysates containing overproduced MutT and NudG binding [α-^32^P](p)ppGpp (2 nM) in the presence of cold competitors and their native substrates (100 μM [each]). Representative DRaCALA spots are shown. 8OdG, 8-oxo-dGTP; 8OG, 8-oxo-GTP; 2OdA, 2-hydroxyl-dATP; 2OA, 2-hydroxyl-ATP. (G) TLC assessment of cleavage products of [α-^32^P]ppGpp (10 nM) determined using purified MutT and NudG (1 μM) in the presence of cold competitors (100 μM) or excess EDTA (25 mM). Samples were incubated at 30°C for 10 min (or 1 h; see Fig. S6D), and reactions were stopped by addition of excess EDTA (25 mM). pGp and ppGpp are indicated.

Upon initial identification, cell lysates containing each of these six proteins specifically bound (p)ppGpp as shown in competition assays (Fig. 5A and B; see also Fig. S6A). In particular, UshA and NudG appeared to have extremely high specificity, as only unlabeled ppGpp and pppGpp, but not the structurally very similar GTP and GDP, could outcompete the bound, radiolabeled (p)ppGpp. In contrast, the radiolabeled (p)ppGpp could be outcompeted to different extents by both unlabeled (p)ppGpp and GTP/GDP, but not by ATP/ADP, off the other four targets. Since PhoA and UshA are periplasmic nonspecific phosphatases that are known to cleave various phosphate-containing chemicals and to be involved in uptake of phosphate from environments, these two proteins were not investigated further in this study.

10.1128/mBio.02188-17.6 FIG S6  Cleavage of (p)ppGpp by MutT, NudG, NadR, and TrmE. (A) Competition assay of cell lysate containing overexpressed PhoA and UshA binding [α-^32^P](p)ppGpp (2 nM) in the presence of cold competitors (100 μM). (B) Binding curves and *K*_*d*_ determination for purified NadR and TrmE binding α-^32^P-labeled GTP, ppGpp, and pppGpp (2 nM [each]). At least two biological replicates were performed. The apparent *K*_*d*_ values corresponding to each protein-ligand interaction are shown. *K*_*d*_ values could not be determined for NadR binding GTP, ppGpp, and pppGpp. (C) Cleavage of [α-^32^P](p)ppGpp (10 nM) (right) and [α-^32^P]GTP (2 nM plus 100 μM cold GTP) (left) by NadR and TrmE in buffer B (containing 40 mM Tris [pH 7.5], 100 mM NaCl, 10 mM MgCl_2_). Ten-micromolar of each protein were used except NadR was used at 20 μM to cleave [α-^32^P]GTP. Cleavage by TrmE was performed also in buffer B containing extra 100 mM KCl (indicated by TrmE + K^+^). Reaction mixtures were incubated at RT for 10 min, stopped by the use of 1.2 M formic acid, and resolved by TLC. (D) Cleavage of [α-^32^P]ppGpp by purified MutT and NudG in buffer B (containing 40 mM Tris [pH 7.5], 100 mM NaCl, 10 mM MgCl_2_). 1 μM of each protein and 100 μM concentrations of cold competitors were used. Reaction mixtures were incubated at 30°C for 1 h, stopped by the use of excess EDTA (50 mM), and resolved by TLC. Download FIG S6, TIF file, 1.7 MB. Copyright © 2018 Zhang et al. 2018 Zhang et al. This content is distributed under the terms of the Creative Commons Attribution 4.0 International license.

The other four proteins (MutT, NudG, TrmE, and NadR) were purified and analyzed further. We found that purified forms of both MutT and NudG failed to bind (p)ppGpp whereas NadR and TrmE showed weak binding of (p)ppGpp (Fig. 5B and C). MutT and NudG are Nudix family enzymes that are known to function in scavenging damaged nucleotides, preferably, 8-oxo-(d)GTP and 2-OH-(d)ATP, respectively, which are produced via the activity of reactive oxygen species (ROS) under conditions of oxidative stress (59). NadR is a multidomain protein with an N-terminal DNA binding domain and with another two domains that are involved in the salvage pathway of NAD biosynthesis (60). TrmE is a GTPase that functions together with MnmG to methylate anticodon wobble position U34 of certain tRNA species. Considering the functions of these proteins and the fact that they either showed weak binding of ppGpp or did not bind at all, it is possible that they degrade (p)ppGpp. Indeed, analysis by TLC showed that both ppGpp and pppGpp were degraded into smaller products by MutT and NudG (Fig. 5D and E). Quantitative analysis indicates that there was some degradation of (p)ppGpp by NadR and TrmE as well. The cleavage of (p)ppGpp by all four proteins was inhibited by EDTA (Fig. 5D and E), suggesting the involvement of magnesium in the cleavage reaction. However, in presence of EDTA, MutT and NudG still did not bind (p)ppGpp, and NadR lost the binding of (p)ppGpp completely (Fig. 5C), indicating the requirement of magnesium for binding (p)ppGpp by these three proteins. In contrast, TrmE still binds (p)ppGpp even when excess EDTA is present, suggesting magnesium-independent binding of (p)ppGpp, similarly to that seen with Der, but also magnesium-dependent weak cleavage activity of (p)ppGpp. As controls, Der and RF3 (data not shown) bound (p)ppGpp irrespective of the presence or absence of magnesium and did not cleave (p)ppGpp (Fig. 5C to E).

The weak activities of (p)ppGpp cleavage and binding by NadR and TrmE were studied further by measuring their binding affinities for (p)ppGpp in the presence of magnesium. NadR showed increasing binding ratios for (p)ppGpp and GTP with increasing concentrations of protein but did not reach a plateau even at 50 µM (Fig. S6B), indicating cleavage of these nucleotides. TrmE, on the other hand, displayed classic saturation binding curves, and apparent *K*_*d*_ values of 1.7 ± 0.5 µM, 2.2 ± 0.9 µM, and 4.6 ± 1.1 µM were determined for ppGpp, pppGpp, and GTP, respectively (Fig. S6B). However, the maximal fractions of binding were low (0.2 on average) compared to those determined for other genuine (p)ppGpp binding proteins (0.4 to 0.6 on average; Fig. 2B and C), suggesting that the low (p)ppGpp cleavage activity of TrmE may contribute to the decreased binding fraction, especially at high concentrations of TrmE (61). Of note, TrmE is an unusual GTPase in that it has a significantly higher intrinsic GTP hydrolysis rate than other bacterial GTPases and the GTP hydrolysis activity is strongly stimulated by potassium ions (62). Therefore, we tested whether potassium could stimulate the hydrolysis of (p)ppGpp. In addition, to probe the degradation products of ppGpp by TrmE and NadR, [α-^32^P]GTP was also used as the substrate and the degradation products were resolved by TLC in parallel with those of [α-^32^P]ppGpp. With potassium ions (100 mM), TrmE (10 µM) showed an increased GTP hydrolysis rate and converted almost all GTP (100 µM) into GDP in 10 min at 25°C, while NadR (20 µM with 100 mM NaCl) had similar GTP hydrolysis activity (Fig. S6C). Both NadR (with sodium) and TrmE (with potassium) converted most of ppGpp (10 nM) into a product that migrated between GTP and GDP. Previously, by using 1.5 M KH_2_PO_4_ (pH 3.4) as the mobile phase, ppGp and pGpp were shown to migrate between GTP and GDP (63). Therefore, this product might represent ppGp or pGpp or both. Together, these data suggest that both NadR and TrmE possess weak (p)ppGpp cleavage activities, such that they may play minor roles in metabolism of (p)ppGpp in *E. coli*.

We focused next on Nudix proteins MutT and NudG. We found that purified MutT and NudG degraded (p)ppGpp (Fig. 5D). To study this further, we first tested whether the native substrates of both MutT and NudG would inhibit the binding of (p)ppGpp by using cell lysates containing overproduced levels of each protein. Indeed, 8-oxo-(d)GTP was able to completely outcompete the MutT-bound (p)ppGpp even better than unlabeled ppGpp and GTP (Fig. 5F). Similarly, 2-OH-(d)ATP was found to be comparable to unlabeled ppGpp in competing away bound (p)ppGpp on NudG, whereas ATP did not compete (Fig. 5F). We thus tested further whether the cleavage of ppGpp by purified proteins would be inhibited by their native substrates. For this, a 1 µM concentration of each protein was incubated with 10 nM [α-^32^P]ppGpp in the presence of 100 µM competitors. MutT cleaved most ppGpp in 10 min at 30°C, and excess EDTA inhibited this activity (Fig. 5G). 8-oxo-(d)GTP totally inhibited the cleavage of [α-^32^P]ppGpp by MutT even after 1 h at 30°C, while both ppGpp and GTP (100 µM) showed very limited inhibitory effects (Fig. S6D). In contrast, NudG showed relatively weak ppGpp cleavage activity compared to MutT. Of note, both experiments using 2-OH-(d)ATP inhibited the cleavage of [α-^32^P]ppGpp slightly better than ppGpp (Fig. 5G; see also Fig. S6D). The terminal products of ppGpp degradation by MutT and NudG are pGp as evidenced by TLC and ultraperformance liquid chromatography-mass spectrometry (UPLC-MS) analysis (Fig. S7), similar to the results seen with Nudix protein Ndx8 from *Thermus thermophilus* (64).

10.1128/mBio.02188-17.7 FIG S7  Cleavage of ppGpp by MutT, NudG, NadR, and TrmE as analyzed by UPLC-MS. 20 μM each of (A) MutT, (B) NudG, (C) NadR, (D) TrmE, and (E) MBP was incubated with 100 μM ppGpp in buffer B (40 mM Tris [pH 7.5], 100 mM NaCl, 10 mM MgCl_2_) for 20 min at room temperature. Samples were snap-frozen on dry ice and analyzed with UPLC-MS (see Materials and Methods for details). The UV data (260 nm wavelength absorption) and the MS chromatograms of the degradative species of ppGpp are shown in the upper and lower parts of each panel, respectively. (F) A table summarizing the relative abundances of the degradative species of ppGpp corresponding to each of the four proteins, with the major species underlined. rt, retention time (min); c(pGp), cyclic 3′,5′-pGp; c(ppGp), cyclic 3′,5′-ppGp. Download FIG S7, TIF file, 1.7 MB. Copyright © 2018 Zhang et al. 2018 Zhang et al. This content is distributed under the terms of the Creative Commons Attribution 4.0 International license.

### MutT and NudG may each constitute alternative (p)ppGpp degradation pathways.

**FIG 6:**
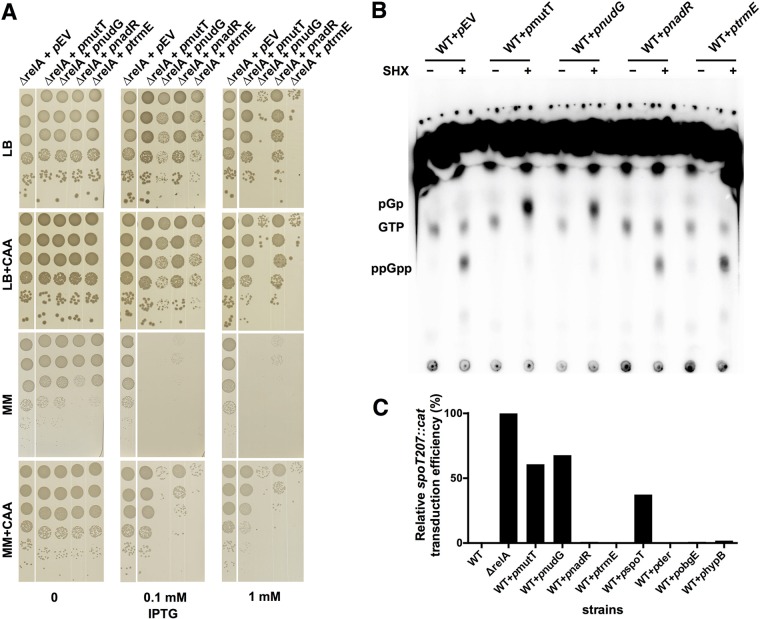
MutT and NudG cleave (p)ppGpp *in vivo*. (A) Plate growth assay of *E. coli ΔrelA* strain bearing extra copies of *mutT*, *nudG*, *nadR*, or *trmE* in pCA24N vector (42) or in empty vector (pEV) on LB and M9 minimal medium (MM) plates with or without 1% (g/ml) Casamino Acids (CAA) and supplemented with IPTG (0, 0.1, or 1 mM). Early-exponential-phase cells were washed three times, adjusted to a CFU count of 4 × 10^7^ with PBS, and serially diluted, and 10 μl was spotted. Plates were incubated at 37°C for 24 h (LB plates and M9 plates with CAA) and 48 h (M9 plates without CAA) before pictures were taken. (B) TLC assessment of (p)ppGpp proteins and their cleavage products produced *in vivo*. Wild-type (WT) MG1655 cells with extra copies of *mutT*, *nudG*, *nadR*, or *trmE* in pCA24N vector or empty vector (pEV) were grown in MOPS minimal medium supplemented with 0.8% (vol/vol) glycerol to the early exponential phase. H_3_^32^PO_4_ (PerkinElmer) (100 μCi/ml) was added, and cells were incubated at 37°C for 1 h. Then, IPTG (1 mM) was added to induce protein expression for 30 min before 0.4 mg/ml serine hydroxamate (SHX) was added to induce amino acid starvation and (p)ppGpp production for 30 min. Samples taken before and after addition of SHX (indicated at the top of the diagram by minus and plus symbols, respectively) were quenched by the use of formic acid (0.333 M final concentration) and resolved by TLC. pGp, GTP, and ppGpp are indicated. (C) Relative efficiencies of transduction of *spoT207*::*cat* into the *E. coli ΔrelA* strain and the wild-type MG1655 strain supplemented with extra copies of *mutT*, *nudG*, *nadR*, *trmE*, *spoT*, *der*, *obgE*, and *hypB* in pNTR vector (68). Three replicates were performed, and the average values of transduction efficiency were normalized to that determined for the *ΔrelA* strain.

We next investigated whether (p)ppGpp hydrolysis by MutT, NudG, NadR, or TrmE could play a role in *E. coli* physiology. As (p)ppGpp is required for growth of *E. coli* in M9 minimal medium (MM) without amino acids (65), we reasoned that the presence of a (p)ppGpp-degrading protein would limit the growth of an *E. coli* strain on MM plates and that addition of Casamino Acids (CAA) would restore the growth defects. To test this proposal, a *relA* strain was transformed with the pCA24N derivatives carrying each of the four corresponding genes. Some cell toxicity was observed when *nudG* and *trmE* were induced with 0.1 mM IPTG on LB or LB-plus-CAA plates, and the level of this inhibition of growth became greater when 1 mM IPTG was used (Fig. 6A). This growth inhibition was probably not related to ppGpp cleavage activity, as ppGpp is not required for growth on LB plates. On MM plates without IPTG, the basal expression levels of all four proteins showed some inhibitory effects on cell growth, but 1% CAA restored the level of growth to that seen with the parental strain with the empty vector (Fig. 6A). Induction with 0.1 mM and 1 mM IPTG produced severely diminished cell growth on MM plates, and CAA restored cell growth to the levels that these strains showed on LB plates, indicating that overexpression of the proteins could indeed perturb cellular levels of ppGpp in all four cases. In particular, overexpression of MutT had no toxic effect on cell growth on LB plates but severely inhibited cell growth on MM plates and the use of 1% CAA completely restored cell growth, suggesting that, in addition to its native substrates, MutT efficiently and specifically degrades (p)ppGpp *in vivo*.

Next, we directly measured (p)ppGpp levels before and after amino acid starvation induced by serine hydroxamate (SHX) (66). As expected, SHX triggered production of (p)ppGpp in the parental wild-type (wt) strain carrying the empty vector, while the strains overexpressing MutT and NudG did not accumulate (p)ppGpp (Fig. 6B). In those two strains, a labeled species corresponding to the expected mobility of pGp appeared, indicating that MutT and NudG convert (p)ppGpp efficiently to pGp *in vivo*. In contrast, the strains overexpressing NadR and TrmE accumulated levels of (p)ppGpp similar to those seen with the parental strain even though NadR overexpression slightly reduced the ppGpp level. Since the potential degradation products of both NadR and TrmE are ppGp and pGpp (Fig. S7), which migrate in a manner very close to that seen with GTP, it would be difficult to pinpoint the degradation products by TLC, especially when the (p)ppGpp cleavage activities of both proteins are weak. These data show that MutT and NudG have strong cleavage activities *in vivo* whereas NadR and TrmE have very weak (p)ppGpp cleavage activities *in vivo*.

The *spoT* gene of a wt *E. coli* strain cannot be deleted (65). In contrast, *spoT* can readily be deleted from a *relA* strain, indicating that the essentiality of *spoT* in the wt strain is due to its (p)ppGpp-hydrolytic activity (65). Previously, a genetic screen in *E. coli* found that extra copies of *mutT* on the high-copy-number pCA24N vector rendered *spoT* nonessential in the wt background (67). Therefore, we tested whether *spoT* would be delectable (by P1 transduction) using a wt strain when extra copies of each of the four test genes were present. For this, we made use of the ASKA mobile collection vectors (68), where each gene of *E. coli* K-12 was cloned on a low-copy-number p15A origin-containing vector, pNTR, under the control of the promoter of pTac/*lacIq*, such that gene expression was IPTG inducible. Of note, two extra residues were present at each end of the *E. coli* genes, with presumably limited effects on protein functions. With the presence of *mutT* or *nudG*, we found that *spoT* deletion mutants were readily obtained even without gene induction, suggesting strong *in vivo* ppGpp degradation activities of both proteins. Furthermore, in the presence of either *mutT* or *nudG* on the plasmid, *spoT* could be deleted at a frequency of 60% to 70% of that of the *relA* control strain (Fig. 6C). In contrast, we were unable to delete *spoT* when either *nadR*-carrying or *trmE*-carrying plasmids were present, consistent with the low levels of ppGpp cleavage activities of NadR and TrmE *in vitro* (Fig. 5D). Taken together, these data suggest that, in addition to SpoT, MutT and NudG may constitute another degradative system for (p)ppGpp in *E. coli*.

### Concluding remarks.

By using DRaCALA in this study, we performed a systematic screening and comparative analysis of (p)ppGpp binding proteins of *E. coli* K-12. Despite some drawbacks of the DRaCALA technique, this study revealed many new targets of (p)ppGpp (Table 1; see also Table S1 in the supplemental material) and provided a global picture of the primary targets of (p)ppGpp in *E. coli* K-12. More importantly, the salvage pathways of GTP biosynthesis and ribosome-related processes were found to be highly conserved targets in *E. coli*, as has also been observed in *Firmicutes* (39, 46). Furthermore, the competitive, transient (fast), and reversible nature of the more potent ppGpp on ribosome-associated GTPases was revealed, and the data explain how bacteria can quickly adapt to various environmental stresses. Last but not least, an alternative degradation pathway for (p)ppGpp was found in *E. coli*. These discoveries, combined with previous reports, form a big picture depicting the action of (p)ppGpp in bacteria under stressful conditions. In the presence of stress, bacteria quickly convert GTP and GDP into (p)ppGpp in amounts comparable to that of the remaining GTP (Fig. 6B) (16). Subsequently, (p)ppGpp binds to RNAP/DksA (in beta- and gammaproteobacteria) or Gmk (in other bacteria) (69) to reprogram global gene expression for stress tolerance/adaptation; on the other hand, (p)ppGpp directly affects GTP biosynthesis and important aspects of ribosome function to directly slow protein production. Therefore, the growth rate is low, thus leading to tolerance of many nutritional and environmental stresses, including those presented by antibiotics.

## MATERIALS AND METHODS

### Bacterial growth conditions and chemicals.

*E. coli* K-12 strains were grown in Luria-Bertani (LB) and MOPS (morpholinepropanesulfonic acid) minimal media supplemented with 0.8% (vol/vol) glycerol at 37°C with agitation (168 rpm). When appropriate, antibiotics were supplemented as indicated in Table S2 in the supplemental material. All chemicals used were purchased from Sigma-Aldrich at the highest grade of purity.

10.1128/mBio.02188-17.9 TABLE S2  Bacterial strains used and constructed. Download TABLE S2, DOCX file, 0.1 MB. Copyright © 2018 Zhang et al. 2018 Zhang et al. This content is distributed under the terms of the Creative Commons Attribution 4.0 International license.

### Strain and plasmid constructions.

Bacterial strains and primers used in this study are listed in Table S2 and Table S3, respectively. For construction of plasmids pET28b-His_6_-*SaDer*, pET28b-His_6_-*SaPrfC*, pET28b-*DerG1*(*2–184*)-His_6_ and pET28b-His_6_-*DerG2KH*(*185–490*), primer pairs YZ185/YZ186, YZ187/YZ188, YZ181/YZ182, and YZ183/YZ184 were used to amplify the *der* and *prfC* genes using *S. aureus* Newman chromosomal DNA as the template and the G1 and G2KH domains of Der using *E. coli* MG1655 chromosomal DNA as the template. The PCR products were digested with NcoI/HindIII and ligated with plasmid pET28b that had been cut with the same enzymes. For construction of pET28b-His_6_.tev-*malE* plasmids, YZ149/YZ150 primer pairs were used to amplify the *malE* gene using vector pMAL-c2x DNA as the template. The PCR products were digested with NcoI/EcoRI and ligated with plasmid pET28b that had been cut with the same enzymes. All plasmids were initially recovered in *E. coli* strain DH5α, and sequences of insertions were confirmed by sequencing (Eurofins Genomics). For protein expression and purification, the plasmids were transformed into *E. coli* strain BL21(DE3), yielding the strains listed in Table S2.

10.1128/mBio.02188-17.10 TABLE S3  Primers used in this study. Download TABLE S3, DOCX file, 0.1 MB. Copyright © 2018 Zhang et al. 2018 Zhang et al. This content is distributed under the terms of the Creative Commons Attribution 4.0 International license.

### Protein expression and purification.

*E. coli* BL21(DE3) (Table S2) and the ASKA collection AG1 strains (42) were used for the expression and purification of all proteins investigated in this study. A 1-liter LB culture of a given strain was grown at 37°C to an optical density at 600 nm (OD_600_) of about 0.3 to 0.5, and protein expression was induced with 0.5 mM IPTG overnight at 18°C. Proteins were purified by nickel-nitriolotriacetic acid (Ni-NTA) affinity chromatography and size exclusion chromatography as previously described (70). Elution fractions containing the protein of interest were pooled and concentrated by using 3-kDa-cutoff centrifugal filters (Amicon). Potential insoluble proteins were removed by centrifugation at 13,400 rpm for 10 min at 4°C, and the concentrations of soluble proteins were determined by the Bradford assay (Bio-Rad). The purity of the purified proteins was assessed in Coomassie-stained 4% to 12% NuPAGE bis-Tris protein gels.

### Synthesis of [α-^32^P](p)ppGpp.

^32^P-labeled pppGpp was synthesized from [α-^32^P]GTP (PerkinElmer) by incubating 125 nM [α-^32^P]GTP with 4 μM purified Rel_Seq_(1–285)-His protein (43) in buffer S (containing 25 mM Tris [pH 9.0], 100 mM NaCl, 15 mM MgCl_2_, and 8 mM ATP) at 37°C for 1 h. The sample was subsequently incubated for 5 min at 95°C to stop synthesis, and the denatured Rel_Seq_(1–285)-His protein was removed by centrifugation at 13,400 rpm for 10 min at 4°C. The supernatant was transferred to a new tube. For synthesis of [α-^32^P]ppGpp, half of the [α-^32^P]pppGpp was transferred to a new tube and 1 μM purified GppA-His protein was added. The sample was incubated at 37°C for 15 min before being heat inactivated for 5 min at 95°C, and the denatured GppA-His protein was removed by centrifugation as described above. The levels of conversion of [α-^32^P]GTP to [α-^32^P]pppGpp and of [α-^32^P]pppGpp to [α-^32^P]ppGpp were determined to be more than 92%, as assessed by thin-layer chromatography (TLC) using 1.5 M KH_2_PO_4_ (pH 3.4) as the mobile phase (39).

### Synthesis of [α-^32^P]GDP.

^32^P-labeled GDP was synthesized from [α-^32^P]GTP by using 20 μM purified TrmE protein in buffer B (containing 40 mM Tris [pH 7.5], 100 mM NaCl, 100 mM KCl, and 10 mM MgCl_2_)–125 nM [α-^32^P]GTP. The sample was incubated at 37°C for 15 min, and the reaction was stopped by adding 1.2 M formic acid (39). The denatured TrmE protein was removed by centrifugation as described above, and the level of conversion of [α-^32^P]GTP to [α-^32^P]GDP was determined by TLC to be 99.8%.

### Differential radial capillary action of ligand assay and screen for (p)ppGpp binding proteins.

DRaCALA screening was performed essentially as described before (39) with minor modifications. Briefly, ASKA collection strains were inoculated into 1.5 ml LB broth with 25 μg/ml chloramphenicol in deep 96-well plates (Greiner) and grown over night at 30°C. IPTG (1 mM) was added the next morning to induce protein expression at 30°C for 6 h. Bacterial cells were collected and frozen at −80°C. To lyse cells, 150 μl buffer B with 2 mM phenylmethylsulfonyl fluoride (PMSF), 40 μg/ml DNase 1, and 0.5 mg/ml lysozyme was used to resuspend cell pellets and then subjected to three freeze-thaw cycles before Benzonase (Sigma) (2.5 U/well) was added to reduce lysate viscosity. A 20-μl volume of lysate was transferred into a 96-well V-bottom plate (Sterilin), incubated at 37°C for 15 min, and placed on ice. A 10-μl volume of [α-^32^P]ppGpp and [α-^32^P]pppGpp premixed at 1:1 was added into each lysate to make a final concentration of 2.5 nM [α-^32^P](p)ppGpp. Mixtures were incubated for 5 min at room temperature (RT) before they were spotted onto a nitrocellulose membrane (Amersham Hybond-ECL; GE Healthcare) using a 96-well pintool (V&P Scientific). Membranes were dried for 10 min at RT, and binding signals were exposed to a BAS IP screen (GE Healthcare) and detected by the use of a Typhoon FLA-7000 PhosphorImager.

For *K*_*d*_ measurements by DRaCALA, 2-fold serial dilutions from the highest possible concentrations of each purified protein were prepared in binding buffer B, and approximately 2 nM concentrations of α-^32^P-labeled ppGpp, pppGpp, GTP, and GDP were added. The mixtures were incubated for 5 min at RT before spotting of 2 µl of the reaction mixtures onto nitrocellulose membranes. The fractions of bound ligand and the apparent *K*_*d*_ values were calculated as previously described (37). For competition assays, purified proteins at the specified concentrations were incubated with 2 nM [α-^32^P]ppGpp or [α-^32^P]pppGpp in the presence of 100 μM competitor nucleotides in binding buffer B. The reaction mixtures were incubated for 5 min at RT, 2 µl was spotted onto nitrocellulose membranes, and the values corresponding to the bound fractions were determined. EDTA was added at excess concentrations as specified for each case.

### *In vitro* [α-^32^P](p)ppGpp and [α-^32^P]GTP cleavage assay.

The purified His_6_-MutT and His_6_-NudG proteins were tested for their relative levels of cleavage of ppGpp compared to those seen with their native substrates. For this, a 1 μM concentration of each protein and a 10 nM concentration of [α-^32^P]ppGpp were used together with 100 μM concentrations of cold competitors [ppGpp, GTP, ATP, 8-oxo-(d)GTP, 2-hydroxyl-(d)ATP] or 50 mM EDTA in buffer B. The samples were incubated at 30°C for 10 min and for 1 h before the reactions were stopped by adding 50 mM EDTA. The cleavage products were resolved by TLC as described above. The purified His_6_-NadR and His_6_-TrmE proteins were tested for their cleavage of both GTP and ppGpp. For cleavage of GTP, 10 μM concentrations of His_6_-MBP, His_6_-TrmE, and 20 μM His_6_-NadR proteins were used with 2 nM [α-^32^P]GTP and 100 μM cold GTP in buffer B. For cleavage of ppGpp, 10 μM concentrations of His_6_-TrmE, His_6_-MBP, and His_6_-NadR proteins were used with 10 nM [α-^32^P]ppGpp in buffer B. Of note, for His_6_-TrmE, another reaction was also performed in buffer B supplemented with 100 mM KCl. The reactions were performed at RT for 10 min and were stopped by adding 17% formic acid. The cleavage products were resolved by TLC as described above.

### UPLC-MS analysis of degradative products of ppGpp by MutT, NudG, NadR, and TrmE.

A 20 μM concentration of each of purified proteins MutT, NudG, NadR, TrmE, and MBP (as a negative control) was incubated with 100 μM ppGpp in buffer B (containing 40 mM Tris [pH 7.5], 100 mM NaCl, and 10 mM MgCl_2_) for 20 min at room temperature. Samples were snap-frozen on dry ice and analyzed with UPLC-MS afterward. All samples and buffers were stored at −80°C, thawed on ice, and kept at 4°C during analysis. For the analysis, a UPLC-quadrupole time of flight (UPLC-qTOF) method (Waters) was used. Analysis conditions were as follows: column, zic-HILIC (Merck Millipore) (150 by 2.1 mm, 3.5 µM pore size, gradient elution, flow rate set to 0.3 ml/min); mobile phase A, 10 mM ammonium acetate (pH 5); mobile phase B, 90% acetonitrile with 10 mM ammonium acetate (pH 5). The gradient was set up as follows: min 0 to 2, 80% B; min 2 to 16, 80% B to 50% B; hold until min 20; injection volume, 4 µl. The detection conditions were as follows: photodiode array detector (PDA), −210 to 400 nm (evaluated at 260 nm); MS, ionization; electrospray ionization (ESI+), scan, 200 to 800 *m*/*z* with lock mass correction. Analytes were identified by their accurate mass and retention time data for a known standard dissolved in used buffer (for the chemicals of the same nominal masses). These standards were either synthesized and their structures confirmed by nuclear magnetic resonance (NMR) (for ppGp, pGpp, and pGp) or purchased (ppGpp was purchased from Jena Biocsience and GTP from Sigma-Aldrich).

### Measurement of (p)ppGpp and cleavage by MutT, NudG, NadR, and TrmE produced *in vivo*.

Overnight cultures of wild-type *E. coli* containing each gene on the pCA24N vector or the empty vector in MOPS minimal medium supplemented with 0.8% (vol/vol) glycerol were diluted 100-fold in the same fresh MOPS minimal medium and grown at 37°C until an OD_600_ of 0.3 to 0.5 was reached. Cells were collected, resuspended to an OD_600_ of 0.1 in the same fresh MOPS medium supplemented with H_3_^32^PO_4_ (PerkinElmer) (100 μCi/ml), and incubated at 37°C and 600 rpm for 1 h (about 1 generation) in a heat block (Eppendorf). A 1 mM concentration of IPTG was added into each culture to induce protein expression for 30 min before 0.4 mg/ml of SHX was added to trigger amino acid starvation and (p)ppGpp production for 30 min. Before and after SHX addition, a 50-μl volume of the cultures was taken out to mix with 10 μl 2 M formic acid, placed on ice for 15 min, and stored at −20°C before being resolved by TLC as described above. Three replicates were performed, and data from only one replicate that was representative of the three are shown.

### **P1 phage transduction of** ***spoT207***::***cat*****.**

P1 phages were prepared from donor strain YZ62 (*ΔrelA spoT207*::*cat*). After testing of the phage titer, 1 ml of prepared P1 phage was used to transduce about 2.5 × 10^9^ CFU of mutant *ΔrelA* (YZ38) and the wild-type MG1655 strain without or with extra copies of genes *mutT*, *nudG*, *nadR*, *trmE*, *spoT*, *der*, *obgE*, and *hypB* on pNTR vectors (68). Transductants were plated on LB plates supplemented with 25 μg/ml chloramphenicol and incubated at 37°C for 48 h before CFU counts were performed. Three replicates were performed for each strain. For each target strain, transduction efficiency was calculated by dividing the number of transductants by the respective CFU counts of the cells used. Relative efficiency levels were calculated by dividing the mean transduction efficiency determined for each strain by the mean efficiency determined for the *ΔrelA* strain.

### Test of the inhibitory effect of MutT, NudG, NadR, and TrmE on cell growth in M9 minimal media.

For tests involving cell growth on plates, LB broth and M9 minimal media with 1.5% (g/ml) agar (Difco) were melted and plates poured without or with 0.1 mM or 1 mM IPTG and without or with CAA (Bacto) (1% [g/ml]). Early-exponential-phase cells grown in LB broth were collected, washed 3 times with phosphate-buffered saline (PBS), and adjusted to a CFU count of 4 × 10^7^. Tenfold serial dilutions were made for each strain, and 10 μl was spotted, dried, and incubated at 37°C. Pictures were taken at 24 h (LB plates and M9 minimal medium plates supplemented with CAA) or at 48 h (M9 minimal medium plates).
