# Supplementary material for: Novel (p)ppGpp Binding and Metabolizing Proteins of Escherichia coli
Source: mBio. 2018 Mar 6;9(2):e02188-17. doi: 10.1128/mBio.02188-17 (PMC5845004; doi:10.1128/mBio.02188-17)
Supplement: TABLE S1 [file mbo001183765st1.docx]

**Table S1. List of proteins that bind or cleave (p)ppGpp.**

| Proteins | Binding (K_d_ or otherwise stated in µM) or cleavage of (p)ppGpp in *E. coli* | Binding or cleavage of (p)ppGpp in other bacteria |
| --- | --- | --- |
| **Purine nucleotide biosynthesis** | | |
| YgdH | 4.0 ± 0.5 (ppGpp, without Mg^2+^)  24.4 ± 4.7 (ppGpp, with 1.5 mM Mg^2+^) 1.59 ± 0.17 (pppGpp, without Mg^2+^)  37.2 ± 6.5 (pppGpp, with 1.5 mM Mg^2+^)  21.4 ± 11.5 (GTP, without Mg^2+^) |  |
| Gpt | 5.2 ± 0.9 (ppGpp); 6.7 ± 0.9 (pppGpp)  IC_50_ = 85 (ppGpp), (1, 2) |  |
| Hpt/HprT | 6.1 ± 0.9 (ppGpp); 6.2 ± 0.8 (pppGpp)  IC_50_ = 85 (ppGpp), (1, 2) | IC_50_ = 11 (ppGpp, *B. subtilis*), (3)  0.37 ± 0.05 (ppGpp); 0.75 ± 0.06 (pppGpp), *S.aureus* (4) |
| Gmk | - | IC_50_ = 20 (ppGpp, *B. subtilis*), (3)  6.02 ± 1.5 (ppGpp); 4.01 ± 1.2 (pppGpp), *S.aureus* (4) |
| GuaB | K_i_ = 30-50; (5) | IC_50_ = 300-500 (ppGpp, *B. subtilis*), (3) |
| PurA | K_i_ = 50, (6); 140, (5) |  |
| Apt | IC_50_ = 1500; (2) |  |
| **Ribosome and translation** | | |
| LepA | ND; stronger binding to ppGpp than pppGpp |  |
| Era | 4.3 ± 0.6 (ppGpp); 21.4 ± 3.6 (pppGpp) | 0.95 ± 0.06 (ppGpp); 3.85 ± 0.56 (pppGpp), *S.aureus* (4) |
| HflX | ND | 0.87 ± 0.15 (ppGpp); 13.63 ± 1.57 (pppGpp), *S.aureus* (4) |
| RsgA | ND | 1.69 ± 0.13 (ppGpp); 10.31 ± 2.59 (pppGpp), *S.aureus* (4) |
| Der | 1.77 ± 0.24 (ppGpp); 6.9 ± 2.9 (pppGpp)  1.3 ± 0.06 (GDP); 13.7 ± 7.1 (GTP) | 3.8 ± 0.5 (ppGpp, **SaDer**, *S. aureus*) 3.5 ± 0.8 (GTP, **SaDer***, S. aureus*) |
| RF3(PrfC) | 0.82 ± 0.09 (ppGpp); 15 ± 5 (pppGpp)  29.3 ± 9.8 (GTP) | No binding of ppGpp by 12 µM of PrfC from *S. aureus* |
| ObgE | 1.8 ± 0.3 (ppGpp); 6.6 ± 0.8 (pppGpp)  K_i_ = 1.6 ± 0.5 (ppGpp) (7) | ND, *B. subtilis* (8) |
| EF-Tu | K_i_ = 60 (ppGpp),(9) |  |
| EF-G | K_i_ = 0.7 and 40 (ppGpp, in the absence and presence of EF-Ts), (9) |  |
| IF2(InfB) | ND (10) |  |
| BipA | ND (11) |  |
| **DNA replication** | | |
| DnaG | IC_50_ = 200, (12) | IC_50_ = 500 (*B. subtilis*), (13) |
| **Transcription** | | |
| RNAP  /DksA | IC_50_ = 15 ± 3, (14) |  |
| **(p)ppGpp homeostasis** | | |
| MutT | Cleavage of (p)ppGpp into pGp | Ndx8 (*T. thermophilus*), (15) |
| NudG | Cleavage of (p)ppGpp into pGp |  |
| TrmE | Cleavage of (p)ppGpp into ppGp/pGpp |  |
| NadR | Cleavage of (p)ppGpp into ppGp/pGpp |  |
| PhoA | Non-specific cleavage of (p)ppGpp into (p)pi |  |
| UshA | Non-specific cleavage of (p)ppGpp into (p)pi |  |
| RelA | Synthesis of and stimulated by (p)ppGpp; (16) |  |
| SpoT | Synthesis and degradation of (p)ppGpp |  |
| GppA | Convert pppGpp to ppGpp; (17) |  |
| **Metabolism** | | |
| HypB | 12.4 ± 3 (ppGpp); 14.8 ± 5.1 (pppGpp) |  |
| LdcI | 1.77 ± 0.24 (ppGpp); 6.9 ± 2.9 (pppGpp), (18) |  |
| LdcC | 0.1-0.5 (ppGpp), (19) |  |
| SpeC | 0.598 ± 0.068 (ppGpp), (19) |  |
| PPX | K_i_ = 200 (ppGpp); K_i_ = 10 (pppGpp), (20) |  |
| YybT  /GdpP | - | K_i_ = 36, (21)  K_i_ = 130, (22) |

a) Color Code as in Table 1 (proteins in red are targets identified prior to and not identified in this study, and those in blue are new targets identified here)

b) -, not applicable; ND, not determined; Binding affinities and cleavage activities are determined in this study, unless otherwise indicated with the according literature wherein it was determined.

References

1. Gallant J, Irr J, Cashel M. 1971. The mechanism of amino acid control of guanylate and adenylate biosynthesis. J Biol Chem 246:5812-6.

2. Hochstadt-Ozer J, Cashel M. 1972. The regulation of purine utilization in bacteria. V. Inhibition of purine phosphoribosyltransferase activities and purine uptake in isolated membrane vesicles by guanosine tetraphosphate. J Biol Chem 247:7067-72.

3. Kriel A, Bittner AN, Kim SH, Liu K, Tehranchi AK, Zou WY, Rendon S, Chen R, Tu BP, Wang JD. 2012. Direct regulation of GTP homeostasis by (p)ppGpp: a critical component of viability and stress resistance. Mol Cell 48:231-41.

4. Corrigan RM, Bellows LE, Wood A, Gründling A. 2016. ppGpp negatively impacts ribosome assembly affecting growth and antimicrobial tolerance in Gram-positive bacteria. Proc Natl Acad Sci U S A 113:E1710-9.

5. Pao CC, Dyess BT. 1981. Effect of unusual guanosine nucleotides on the activities of some Escherichia coli cellular enzymes. Biochim Biophys Acta 677:358-62.

6. Stayton MM, Fromm HJ. 1979. Guanosine 5'-diphosphate-3'-diphosphate inhibition of adenylosuccinate synthetase. J Biol Chem 254:2579-81.

7. Persky NS, Ferullo DJ, Cooper DL, Moore HR, Lovett ST. 2009. The ObgE/CgtA GTPase influences the stringent response to amino acid starvation in Escherichia coli. Mol Microbiol 73:253-66.

8. Buglino J, Shen V, Hakimian P, Lima CD. 2002. Structural and biochemical analysis of the Obg GTP binding protein. Structure 10:1581-92.

9. Rojas AM, Ehrenberg M, Andersson SG, Kurland CG. 1984. ppGpp inhibition of elongation factors Tu, G and Ts during polypeptide synthesis. Mol Gen Genet 197:36-45.

10. Milon P, Tischenko E, Tomsic J, Caserta E, Folkers G, La Teana A, Rodnina MV, Pon CL, Boelens R, Gualerzi CO. 2006. The nucleotide-binding site of bacterial translation initiation factor 2 (IF2) as a metabolic sensor. Proc Natl Acad Sci U S A 103:13962-7.

11. Fan H, Hahm J, Diggs S, Perry JJ, Blaha G. 2015. Structural and Functional Analysis of BipA, a Regulator of Virulence in Enteropathogenic Escherichia coli. J Biol Chem 290:20856-64.

12. Maciag M, Kochanowska M, Lyzen R, Wegrzyn G, Szalewska-Palasz A. 2010. ppGpp inhibits the activity of Escherichia coli DnaG primase. Plasmid 63:61-7.

13. Rymer RU, Solorio FA, Tehranchi AK, Chu C, Corn JE, Keck JL, Wang JD, Berger JM. 2012. Binding mechanism of metalNTP substrates and stringent-response alarmones to bacterial DnaG-type primases. Structure 20:1478-89.

14. Ross W, Sanchez-Vazquez P, Chen AY, Lee JH, Burgos HL, Gourse RL. 2016. ppGpp Binding to a Site at the RNAP-DksA Interface Accounts for Its Dramatic Effects on Transcription Initiation during the Stringent Response. Molecular Cell 62:811-823.

15. Ooga T, Ohashi Y, Kuramitsu S, Koyama Y, Tomita M, Soga T, Masui R. 2009. Degradation of ppGpp by nudix pyrophosphatase modulates the transition of growth phase in the bacterium Thermus thermophilus. J Biol Chem 284:15549-56.

16. Shyp V, Tankov S, Ermakov A, Kudrin P, English BP, Ehrenberg M, Tenson T, Elf J, Hauryliuk V. 2012. Positive allosteric feedback regulation of the stringent response enzyme RelA by its product. EMBO Rep 13:835-9.

17. Keasling JD, Bertsch L, Kornberg A. 1993. Guanosine pentaphosphate phosphohydrolase of Escherichia coli is a long-chain exopolyphosphatase. Proc Natl Acad Sci U S A 90:7029-33.

18. Kanjee U, Gutsche I, Alexopoulos E, Zhao BY, El Bakkouri M, Thibault G, Liu KY, Ramachandran S, Snider J, Pai EF, Houry WA. 2011. Linkage between the bacterial acid stress and stringent responses: the structure of the inducible lysine decarboxylase. Embo Journal 30:931-944.

19. Kanjee U, Gutsche I, Ramachandran S, Houry WA. 2011. The enzymatic activities of the Escherichia coli basic aliphatic amino acid decarboxylases exhibit a pH zone of inhibition. Biochemistry 50:9388-98.

20. Kuroda A, Murphy H, Cashel M, Kornberg A. 1997. Guanosine tetra- and pentaphosphate promote accumulation of inorganic polyphosphate in Escherichia coli. J Biol Chem 272:21240-3.

21. Rao F, See RY, Zhang D, Toh DC, Ji Q, Liang ZX. 2010. YybT is a signaling protein that contains a cyclic dinucleotide phosphodiesterase domain and a GGDEF domain with ATPase activity. J Biol Chem 285:473-82.

22. Corrigan RM, Bowman L, Willis AR, Kaever V, Grundling A. 2015. Cross-talk between Two Nucleotide-signaling Pathways in Staphylococcus aureus. Journal of Biological Chemistry 290:5826-5839.
