# Supplementary material for: Novel (p)ppGpp Binding and Metabolizing Proteins of Escherichia coli
Source: mBio. 2018 Mar 6;9(2):e02188-17. doi: 10.1128/mBio.02188-17 (PMC5845004; doi:10.1128/mBio.02188-17)
Supplement: TABLE S2 [file mbo001183765st2.docx]

**Table S2: Bacterial strains used and constructed**

| **Strain** | **Relevant features** | **Reference** |
| --- | --- | --- |
| DH5α | Standard cloning strain | Stratagene |
| BL21(DE3) | *E. coli* strain used for protein expression | Novagen |
| YZ5 | XL1 Blue pET28b-GppA-His_6_; KanR | (1) |
| YZ6 | BL21(DE3) pET28b-GppA-His_6_; KanR | (1) |
| YZ7 | DH5α pENH385-Rel_Seq_-His_6_; AmpR | (2) |
| YZ8 | BL21(DE3) pENH385-Rel_Seq_-His_6_; AmpR | (2) |
| YZ9 | DH5α pET28b; KanR | Laboratory stock |
| YZ14 | DH5α pMAL-c2x; AmpR | Laboratory stock |
| YZ37 | MG1655 | Laboratory stock |
| YZ38 | MG1655 *ΔrelA* | This study |
| YZ62 | MG1655 *ΔrelA spoT207::cat*; CamR | This study |
| YZ64 | MG1655 *spoT207::cat pNTR-SD-mutT*; AmpR;CamR | This study |
| YZ66 | MG1655 *spoT207::cat pNTR-SD-nudG*; AmpR;CamR | This study |
| YZ78 | MG1655 pCA24N-*mutT*; CamR | This study |
| YZ79 | MG1655 pCA24N-*nudG*; CamR | This study |
| YZ80 | MG1655 pCA24N; CamR | This study |
| YZ81 | MG1655 *ΔrelA* pCA24N; CamR | This study |
| YZ161 | DH5α pET28b-His_6._tev-MalE; KanR | This study |
| YZ167 | BL21(DE3) pET28b-His_6._tev-MalE; KanR | This study |
| YZ188 | MG1655 pCA24N-*nadR*; CamR | This study |
| YZ189 | MG1655 pCA24N-*trmE*; CamR | This study |
| YZ54 | MG1655 pNTR-*mutT*; AmpR | This study |
| YZ55 | MG1655 pNTR-*nudG*; AmpR | This study |
| YZ192 | MG1655 pNTR-*trmE*; AmpR | This study |
| YZ193 | MG1655 pNTR-*hypB*; AmpR | This study |
| YZ194 | MG1655 pNTR-*obgE*; AmpR | This study |
| YZ195 | MG1655 pNTR-*der*; AmpR | This study |
| YZ196 | MG1655 pNTR-*spoT*; AmpR | This study |
| YZ197 | MG1655 pNTR-*nadR*; AmpR | This study |
| YZ204 | MG1655 *ΔrelA* pCA24N-*mutT*; CamR | This study |
| YZ205 | MG1655 *ΔrelA* pCA24N-*nudG*; CamR | This study |
| YZ206 | MG1655 *ΔrelA* pCA24N-*nadR*; CamR | This study |
| YZ207 | MG1655 *ΔrelA* pCA24N-*trmE*; CamR | This study |
| YZ226 | DH5α pET28b-DerG1(2-184)-His_6_; KanR | This study |
| YZ227 | DH5α pET28b-His_6_-DerG2KH(185-490); KanR | This study |
| YZ228 | DH5α pET28b-His_6_-SaDer; KanR | This study |
| YZ229 | DH5α pET28b-His_6_-SaPrfC; KanR | This study |
| YZ230 | BL21(DE3) pET28b-DerG1(2-184)-His_6_; KanR | This study |
| YZ231 | BL21(DE3) pET28b-His_6_-DerG2KH(185-490); KanR | This study |
| YZ232 | BL21(DE3) pET28b-His_6_-SaDer; KanR | This study |
| YZ233 | BL21(DE3) pET28b-His_6_-SaPrfC; KanR | This study |

References

1. Corrigan RM, Bellows LE, Wood A, Gründling A. 2016. ppGpp negatively impacts ribosome assembly affecting growth and antimicrobial tolerance in Gram-positive bacteria. Proc Natl Acad Sci U S A 113:E1710-9.

2. Hogg T, Mechold U, Malke H, Cashel M, Hilgenfeld R. 2004. Conformational antagonism between opposing active sites in a bifunctional Re1A/SpoT homolog modulates (p)ppGpp metabolism during the stringent response. Cell 117:57-68.
