# Supplementary material for: Novel (p)ppGpp Binding and Metabolizing Proteins of Escherichia coli
Source: mBio. 2018 Mar 6;9(2):e02188-17. doi: 10.1128/mBio.02188-17 (PMC5845004; doi:10.1128/mBio.02188-17)
Supplement: TABLE S3 [file mbo001183765st3.docx]

**Table S3: Primers used in this study**

| **Number** | **Name** | **Sequence^a)^** |
| --- | --- | --- |
| YZ34 | pCA24Nf | GGCCCTTTCGTCTTCACCTC |
| YZ35 | pCA24Nr | GGCAACCGAGCGTTCTGAAC |
| YZ72 | pNTRSD-seqF | GGAAACAGAATTAAGCTATG |
| YZ73 | pNTRSD-seqR | GTATCAGGCTGAAAATCTTC |
| YZ149 | NcoI-his.tev.malE-F | CATGCCATGGGACACCACCACCACCACCACCATGAAAACCTGTATTTCCAGGGTAAAATCGAAGAAGGTAAACT |
| YZ150 | EcoRI-malE-R | CGGAATTCTTACTTGGTGATACGAGTCTGCGCGTCTTTCAGGG |
| YZ181 | NcoI-DerG1.his-F | CATGCCATGGTACCTGTGGTCGCGCTTGT |
| YZ182 | HindIII-DerG1.his-R | CCCAAGCTTTTAGTGGTGGTGGTGGTGGTGCGCTTCAAATTGCGCCCAGT |
| YZ183 | NcoI-his.DerG2KH-F | CATGCCATGGCACACCACCACCACCACCACGAAGAGAACGGCGAAGAAGA |
| YZ184 | HindIII-his.DerG2KH-R | CCCAAGCTTTTATTTATTTTTCTTGATGTGCT |
| YZ185 | NcoI-his.SaDer-F | CATGCCATGGCACACCACCACCACCACCACACTAAACCTATAGTAGCTAT |
| YZ186 | HindIII-his.SaDer-R | CCCAAGCTTTTAATTTCTCTTTCGAGCTA |
| YZ187 | NcoI-his.SaPrfC-F | CCATGGCACACCACCACCACCACCACAACTTAAAGCAAGAAGTTGA |
| YZ188 | HindIII-his.SaPrfC-R | CCCAAGCTTTTAAAGTAAACTATACAATT |

1. Restriction sites in DNA oligonucleotides are underlined
